# Evidence-based management approaches for patients with severe chronic obstructive pulmonary disease (COPD): A practice review

**DOI:** 10.1177/02692163221079697

**Published:** 2022-03-20

**Authors:** Yu Fu, Emma J Chapman, Alison C Boland, Michael I Bennett

**Affiliations:** 1 Population Health Sciences Institute, Newcastle University, Newcastle upon Tyne, UK; 2 Academic Unit of Palliative Care, Leeds Institute of Health Sciences, University of Leeds, Leeds, UK; 3 Department of Respiratory Medicine, St James’s University Hospital, Leeds, UK

**Keywords:** COPD, evidence-based practice, clinical guidelines, review, exacerbations, palliative medicine

## Abstract

**Background:**

Patients with chronic obstructive pulmonary disease (COPD) face limited treatment options and inadequate access to palliative care.

**Aim:**

To provide a pragmatic overview of clinical guidelines and produce evidence-based recommendations for severe COPD. Interventions for which there is inconsistent evidence to support their use and areas requiring further research were identified.

**Design:**

Practice review of guidelines supported by scoping review methodology to examine the evidence reporting the use of guideline-recommended interventions.

**Data sources:**

An electronic search was undertaken in MEDLINE, EMBASE, PsycINFO, CINAHL and The Cochrane Database of Systematic Reviews, complemented by web searching for guidelines and publications providing primary evidence (July 2021). Guidelines published within the last 5 years and evidence in the last 10 years were included.

**Results:**

Severe COPD should be managed using a multidisciplinary approach with a holistic assessment. For stable patients, long-acting beta-agonist/long-acting muscarinic antagonist and pulmonary rehabilitation are recommended. Low dose opioids, self-management, handheld fan and nutritional support may provide small benefits, whereas routine corticosteroids should be avoided. For COPD exacerbations, systematic corticosteroids, non-invasive ventilation and exacerbation action plans are recommended. Short-acting inhaled beta-agonists and antibiotics may be considered but pulmonary rehabilitation should be avoided during hospitalisation. Long term oxygen therapy is only recommended for patients with chronic severe hypoxaemia. Short-acting anticholinergic inhalers, nebulised opioids, oral theophylline or telehealth are not recommended.

**Conclusions:**

Recommended interventions by guidelines are not always supported by high-quality evidence. Further research is required on efficacy and safety of inhaled corticosteroids, antidepressants, benzodiazepines, mucolytics, relaxation and breathing exercises.

**What is already known about the topic?**

- Patients with severe COPD face frequent exacerbations, hospitalisation and shortened survival but limited access to palliative care.
- Neither standardised guidelines nor synthesis is available on recommendations for severe COPD.
- Many clinicians are unsure how to best treat or support severe COPD.

**What this paper adds?**

- Not all guidelines are developed based on robust evidence.
- There is a lack of high-quality studies focussing on patients with severe COPD.
- Evidence supports the multidisciplinary approach with combination inhaled therapies.
- Practical recommendations are made for the management of stable COPD and COPD exacerbations respectively balancing the efficacy and harms.

**Implications for practice, theory or policy**

- Severe COPD should be managed by a multidisciplinary approach with a holistic assessment.
- Stable patients will benefit from combination inhaled therapies such as long-acting beta-agonist/long-acting muscarinic antagonist are preferred over monotherapies, low dose opioids and non-pharmacological interventions such as self-management, pulmonary rehabilitation, a handheld fan can provide small benefits, but routine corticosteroids should be avoided.
- For COPD exacerbations, systematic corticosteroids, non-invasive ventilation and exacerbation action plans are recommended, short-acting inhaled beta-agonists and antibiotics may provide benefits but pulmonary rehabilitation should be avoided during hospitalisation.
- Long term oxygen therapy is only recommended for patients with chronic severe hypoxaemia.
- Short-acting anticholinergic inhalers, nebulised opioids, oral theophylline or telehealth are not recommended.
- Further research is required to investigate the efficacy and safety of inhaled corticosteroids, antidepressants, benzodiazepines, mucolytics, relaxation and breathing exercises in patients with severe COPD.

## Introduction

Chronic obstructive pulmonary disease (COPD) is a leading cause of death worldwide.^1^ Poorly managed severe COPD (forced expiratory volume in 1 s (FEV1) <50% predicted^2^) may lead to frequent exacerbations, hospitalisation and shortened survival.^3^ Patients experience a similar symptom burden to those with lung cancer with unmet palliative care needs,^4^ yet have limited access and lower uptake of palliative care often due to unpredictable prognosis.^5^

Palliative care has predominately focussed on malignant disease, neither standardised guidelines nor synthesis is available for end of life of non-malignant diseases or the management of patients with severe COPD concerning early palliative care measures. In addition, not all guidelines are evidence-based. Although a growing body of research supports early palliative care approaches for better health outcomes,^6^ many health systems are not set up to provide trained palliative care clinicians to patients and many clinicians experience difficulties in when, how and what to initiate palliative care,^7^ leading to inadequate provision of palliative care for COPD patients. Palliative care is shown only frequently to be provided within the last few weeks of life in patients with severe COPD instead of their last year of life, and it is more likely for patients to receive it if a lung cancer co-diagnosis exists.^5^ We aim to (1) provide a critical yet pragmatic overview of clinical guidelines, (2) produce evidence-based recommendations for practice and (3) identify gaps that require further research.

## Methods

This practice review is to provide an overview of current guidelines with a supporting evidence based summary of recommendations drawn from the available evidence. National and international guidelines that described the management of COPD in adults were searched and examined and interventions recommended by guidelines were identified. A scoping review was then undertaken to provide evidence base supporting the recommendation of these interventions and identify areas where the evidence-base is lacking, and further research is required. A series of searches in MEDLINE, EMBASE, PsycINFO, CINAHL and The Cochrane Database of Systematic Reviews were made using a range of keywords and subject headings (July 2021, Supplemental Material 1). References lists of guidelines and supporting evidence were scrutinised. Guideline and supporting study eligibility are shown in Table 1. It is worth noting that asthma and COPD are different disorders although they may coexist, in which asthma guidelines for pharmacotherapy plus pharmacological and non-pharmacological approaches for COPD should be used.^2^

**Table 1.** Eligibility of guideline and supporting study.

Guidelines were included if: • Being national or international guidelines • COPD management in adults • Published in the last 5 years • English language	
Supporting studies were included if: • Reporting effects and/or harms of guideline-recommended interventions • Adults with severe COPD or in the palliative care phase • Published in the last 10 years • English language	Supporting studies were excluded if: • Focussing on disease-modifying interventions such as smoking cessation or lung volume reduction surgery • Opinion pieces • Protocols or no access to the full text

Identified citations were exported to EndNote for deduplication and selected after independent screening of titles and abstracts by two researchers (YF, EJC) any discrepancies resolved by discussion with a third author. Results are presented according to Preferred Reporting Items for Systematic reviews and Meta-Analyses extension for Scoping Reviews was followed^8^ (Supplemental Material 2).

Each intervention was allocated to a practice recommendation of ‘Do’, ‘Don’t’ or ‘Don’t know’ based on consistency and quality of the supporting empirical evidence. The strength of recommendation was rated as ‘strong’, ‘moderate’ and ‘tentative’ balancing benefits and harms according to criteria listed in Table 2.

**Table 2.** Category of practice recommendation and strength of evidence.

Recommendation category	
Do	A rich body of high quality published evidence for effectiveness in managing severe COPD
Do not	Evidence shows that the intervention is ineffective in managing advanced COPD
Don’t know	No/unclear evidence for effectiveness in managing severe COPD *Or* Lack of high-quality evidence for effectiveness in managing severe COPD *Or* High-quality published evidence to support its use in other medical conditions rather than severe COPD
Strength of evidence	
Strong	A large and consistent body of evidence such as a systematic review
Moderate	Solid empiric evidence from one or more published studies
Tentative	Limited empiric evidence

## Results

Twenty four guidelines were identified but 18 were included as six guidelines used other guidelines as the prime evidence base. A summary of guidelines with recommendations is presented in Table 3. The strength of each recommendation is presented in Table 4. Evidence used to support practical recommendations is presented in Supplemental Material 3.

**Table 3.** Summary of guidelines and recommendations.

Guidelines reviewed	Multidisciplinary approach	Assessment	Pharmacological management	NIV	Long term oxygen therapy	Self-management	Pulmonary rehabilitation	Telehealth	Psychological care	Supportive care
Global Strategy for the Diagnosis, Management, and Prevention of Chronic Obstructive Pulmonary Disease (2020 report)										
Chronic obstructive pulmonary disease in over 16s: diagnosis and management NICE guideline (2018)										
Roflumilast for treating chronic obstructive pulmonary disease Technology appraisal guidance (2017)										
Scottish Palliative Care Guidelines – Breathlessness NHS Scotland (2019)										
All Wales COPD Management and Prescribing Guideline (2020)										
Pharmacologic Management of Chronic Obstructive Pulmonary Disease: An Official American Thoracic Society Clinical Practice Guideline (2020)										
Prevention of COPD exacerbations: a European Respiratory Society/American Thoracic Society guideline (2017)										
Management of COPD exacerbations: a European Respiratory Society/American Thoracic Society guideline (2017)										
Canadian Thoracic Society Clinical Practice Guideline on pharmacotherapy in patients with COPD – 2019 update of evidence (2019)										
Spanish COPD Guidelines (GesEPOC) 2017. Pharmacological Treatment of Stable Chronic Obstructive Pulmonary Disease (2017)										
Health Care Guideline: Diagnosis and Management of Chronic Obstructive Pulmonary Disease (2016)										
Managing Malnutrition in COPD Including a pathway for the appropriate use of Oral Nutritional Supplements (ONS) to support community healthcare professionals (2020)										
British Thoracic Society guideline for the use of long-term macrolides in adults with respiratory disease (2020)										
COVID-19 rapid guideline: community-based care of patients with chronic obstructive pulmonary disease (COPD) NICE guideline (2020)										
Chronic obstructive pulmonary disease (acute exacerbation): antimicrobial prescribing NICE guideline (2018)										
Long-Term Noninvasive Ventilation in Chronic Stable Hypercapnic Chronic Obstructive Pulmonary Disease: An Official American Thoracic Society Clinical Practice Guideline (2020)										
European Respiratory Society guidelines on long-term home non-invasive ventilation for management of COPD (2019)										
Home Oxygen Therapy for Adults with Chronic Lung Disease: An Official American Thoracic Society Clinical Practice Guideline										

**Table 4.** Practice recommendations and strength of evidence.

Practice recommendation	Strength of evidence
***Do***	
Multidisciplinary approach and assessment	Strong
Pharmacological management	
LABA	Strong
LAMA	Moderate
SABA/SAMA	Moderate
LABA/LAMA	Strong
ICS/LABA	Tentative
ICS/LABA/LAMA	Tentative
Systemic corticosteroids for acute exacerbations	Moderate
Opioids	Tentative
Antibiotics	Tentative
LTOT (PaO_2_ ⩽ 7.3 kPa)	Moderate
NIV	
Acute hypercapnic respiratory failure	Moderate
Home NIV for chronic stable hypercapnic	Tentative
Self-management	Moderate
PR	Moderate
CBT-based psychological therapies	Tentative
Supportive care	
Fan blowing air	Moderate
Nutritional support	Moderate
***Do not***	
Pharmacological management	
SAMA	Tentative
Routine use of systemic corticosteroids	Moderate
Nebulised opioids	Moderate
Theophylline	Moderate
Routine use of PDE₄	Tentative
PR during hospitalisation	Strong
Telehealth	Moderate
***Don’t know***	
Medications	
SABA	
Routine use of ICS	
Antidepressants	
Benzodiazepines	
Routine use of mucolytic	
Relaxation	
Breathing exercises	

### Do

#### Multidisciplinary management and assessment

A multidisciplinary approach is strongly recommended. Multidisciplinary and multi-treatment improve breathlessness, dysponea, fatigue, disease impact and activity and reduce hospital admissions and hospitalisation days. Mixed evidence on emotional and symptom mastery are observed.^9,10^ A randomised controlled trial (RCT) finds early integration of palliative care increases survival rate for patients.^11^

Holistic assessment is recommended and severity of airflow limitation should be indicated. A comprehensive assessment of symptoms measuring breathlessness by MRC scale^12^ and symptom burden by COPD Assessment Test (CAT),^13^ exercise capacity by the 6-minute walking distance,^14,15^ oxygen saturation,^16^ history of hospital admissions and exacerbations^17,18^ should be undertaken. Comorbidities^19^ and Alpha-1 antitrypsin deficiency^20^ should be assessed. Blood eosinophil counts^21^ should also be used in combination with clinical assessment of exacerbation risk to predict the treatment effect of inhaled corticosteroids containing regimes.

#### Pharmacological management

##### Beta-agonist

Cochrane reviews provide moderate to high-quality evidence that long-acting beta-agonist (LABA) is associated with improvement in lung function, exacerbations and quality of life (QoL), but no impact on mortality.^22,23^

##### Anticholinergics

Long-acting muscarinic antagonist (LAMA) are recommended to reduce exacerbation.^2,24^ Tiotropium reduces risk of exacerbations (and related hospitalisations) and clinical deterioration, with an increase in clinical improvement and QoL.^25–27^ An associated risk of increased mortality is observed in patients with soft mist inhaler compared with placebo^25^ but no difference in deaths with ipratropium bromide.^26^

##### Combination inhaled therapy

Guidelines^2,28–32^ recommend the use of a combination of medications over monotherapies with different mechanisms that may increase overall efficacy and lower adverse effects.

##### Short acting beta agonists (SABA)/short-acting anticholinergic (SAMA)

Recent evidence is limited on the use of SABA/SAMA but suggested a potential effect on exacerbation, patients also showed a preference for using Respimat inhaler.^34^

##### LABA/LAMA

Long term efficacy and safety of LABA/LAMA are demonstrated across multiple trials for bronchodilation and dyspnoea^33–36^ in moderate and severe patients, leading to a strong Do. Cochrane reviews further suggest a small improvement in lung function and QoL with LABA/LAMA but inconsistent effects on exacerbations which may be caused by heterogeneity.^37,38^ Compared with nebulised SABA/SAMA, effect on FEV1 is not superior over 6h with a slower reaching of lower peak FEV1 in dry powder with LABA/LAMA.^39^

##### ICS/LABA

Cochrane reviews suggest that ICS/LABA reduces exacerbations and potentially improves mortality, lung function, symptoms and QoL, compared with ICS, LABA or usual care.^40–42^ However an increased risk of pneumonia is noted in patients on combined inhalers.^41^

##### ICS/LABA/LAMA

Triple therapy decreases the risk of exacerbations and improves QoL in patients with and without a history of exacerbations in the past year, however it increases the risk of pneumonia compared with LABA/LAMA.^43–45^ A Cochrane review provides good quality evidence ICS/LABA/tiotropium has a small effect on lung function and QoL compared with tiotropium alone. No conclusion could be drawn on impact on exacerbations due to heterogeneity between trials.^46^

##### Systemic corticosteroids in acute exacerbations

There is high-quality evidence to recommend systemic corticosteroids for acute exacerbations, which improve symptoms and lung function, reduce treatment failure and relapse and duration of hospitalisation.^47^ Short courses of oral corticosteroids are in favour leading to a decrease in pneumonia admissions and all-cause mortality.^48^ Risk of adverse effects such as hyperglycaemia is increased.

##### Opioids

A tentative recommendation is made for low dose (10–30 mg daily) opioids.^49^ Reviews suggest a reduction in breathlessness with systemic opioids in stable severe COPD without effect on exercise capacity.^50^ Opioid-related adverse events including nausea, vomiting and constipation are common but often self-limiting on withdrawal.^49^ Trials suggest improvement in patient’s breathlessness with low dose opioids.^51,52^ Future studies to investigate longer-term benefits and risks are needed.

##### Antibiotics

Antibiotics are tentatively recommended concerning antibiotic resistance and other adverse events.^2,24,28,32,53–57^ A Cochrane review finds small effects in outpatients on treatment failure, no effect in inpatients with severe exacerbations, but a large effect among patients in the intensive care unit patients. Routine use of macrolides shows a clinically meaningful reduction in exacerbations.^58–60^ Serious adverse events are reported including hearing impairment, long QTc and tinnitus.^59,60^

#### Long term oxygen therapy (LTOT)

Oxygen for more than 15 h per day is recommended for patients with chronic severe hypoxaemia (a PaO_2_ ⩽ 7.3 kPa).^61^ Cochrane reviews and trials suggest survival benefits in patients with severe hypoxaemia^62^ but improve breathlessness only during exercise in mildly hypoxaemic and non-hypoxaemic patients.^63^ No effect is observed on QoL or hospitalisation, although serious adverse events are rare.^63–65^ For patients with advanced life-limiting diseases with PaO_2_ > 7.3 kPa, palliative oxygen does not improve breathlessness relief compared with air^65,66^ although studies are undertaken with a majority of cancer patients. Regular assessment and support should be undertaken to ensure compliance, along with monitoring for hypercapnia and optimal oxygen saturation.

#### Non-invasive ventilation (NIV)

NIV is recommended for patients with acute hypercapnic respiratory failure in patients hospitalised for acute exacerbations.^67^ A Cochrane review^68^ with moderate evidence suggests NIV decreases risk of mortality, needing endotracheal intubation and complications and reduces length of hospital stay. It is also associated with improved pH and partial pressure of oxygen. For stable patients, a tentative recommendation is made due to weak evidence of efficacy of long term home NIV, showing a small effect on QoL, possible fewer hospitalisations but no survival benefit.^69,70^ Mild but manageable adverse effects are common.^71^

#### Self-management

Self-management is often delivered in an interactive process covering multi interventions including an action plan for acute exacerbation. Cochrane reviews^72,73^ with low to moderate quality evidence show self-management reduces respiratory-related hospital admissions and improves QoL and dyspnoea. The use of COPD exacerbation action plans with a single brief educational component, in conjunction with ongoing support, is found to reduce in-hospital healthcare utilisation and increases treatment of COPD exacerbations with corticosteroids and antibiotics.^74^ Heterogeneity in interventions components, duration and outcome measures requires further studies to inform the most optimised content.

#### Pulmonary rehabilitation

Pulmonary rehabilitation has a strong focus on physical exercise and has benefits on dyspnoea, fatigue, emotional function and symptom mastery, and QoL in studies of very low to moderate quality.^75^ In patients with a recent hospitalisation for an acute exacerbation, moderate to large effects are observed on QoL and exercise capacity. Pulmonary rehabilitation also reduces readmissions but does not improve mortality, but results are heterogeneous.^76^ However trials suggest mixed findings of the effect on long-term clinical outcomes.^77,78^ Further evidence is needed to determine the effective components and format.

#### Cognitive Behavioural Therapy (CBT) based psychological therapies

A Cochrane review with low-quality evidence demonstrates a small effect on depression.^79^ Other studies suggest a small impact on anxiety,^80–82^ but there is one review that reports a non-significant effect of using CBT on patients’ emotional status.^83^ High-quality studies are needed to confirm effectiveness of psychological therapies and to identify comparative effectiveness.

#### Supportive care

##### Handheld fan

Small trials suggest use of a handheld fan directed to the face may improve patients’ breathlessness.^84–87^ Patients show favour in routine use considering low costs and an absence of risks.

##### Nutritional support

Moderate-quality evidence supports an association between supplementation and weight gain, respiratory muscle strength, walking and QoL with larger effects if malnourished.^88,89^ A review using individual patient data suggests vitamin D reduces moderate and severe exacerbations in patients with baseline 25-hydroxyvitamin D levels <25 nmol/L.^90^ Common adverse effects may include bloating, leading to low supplementation adherence.

### Do not

#### SAMA

A Cochrane review with moderate to high quality of evidence suggests ipratropium bromide monotherapy is less effective in improving lung function, hospital admissions, exacerbations and QoL, compared to tiotropium.^26^

#### Routine use of systemic corticosteroids

A Cochrane review finds that longer courses (10–14 days) of systemic corticosteroids (both oral and intravenous) do not reduce the risk of treatment failure, relapse or time to re-exacerbation compared with shorter courses in severe or very severe patients. No difference in the likelihood of adverse events is found, including hyperglycaemia, hypertension, gastrointestinal tract bleeding and symptoms of congestive heart failure or ischaemic heart disease.^91^ A moderate Do not recommendation is made due to lack of efficacy and potential toxicity.

#### Nebulised opioids

A Cochrane review with low-quality of evidence finds no evidence that nebulised opioids are more effective than placebo although a wide range of doses is delivered (1–50 mg).^92^ Another review demonstrates a lack of benefit on exercise capacity and breathlessness. Common adverse effects include lightheadedness, dizziness and mild nausea.^50^ A moderate Do not recommendation is made due to lack of clinical benefit in studies of poor quality.

#### Theophylline

Recent research is limited and not in favour of theophylline concerning the likelihood of serious adverse events and no additional benefit in reducing exacerbations.^93,94^ A possible increased risk of overall exacerbations is reported.^93^

#### Routine use of phosphodiesterase-4 (PDE₄)

Routine use of PDE₄ should be avoided given the small benefit but a range of adverse events. A Cochrane review^95^ suggests PDE₄ is associated with a small improvement in FEV1, QoL and exacerbation frequency in moderate to very severe patients. No effects are observed on symptoms or exercise tolerance. However patients experience increased depressive symptoms and insomnia, weight loss and diarrhoea, nausea and vomiting.

#### Pulmonary rehabilitation during hospitalisation

When Pulmonary rehabilitation is initiated early during acute admission for exacerbation, no consistent improvement is observed on mortality, hospital readmission, QoL or exercise capacity.^55^ Trials demonstrate no difference in readmission or recovery of physical function but a slightly higher mortality rate.^96^ Higher rates of adverse events relating to fall, blood pressure, heart rate although not severe are reported.^55,97^

#### Telehealth

Telehealth is remote delivery of healthcare or monitoring disease,^98^ which has no impact on mortality but a possible improvement on QoL and emergency department and hospital attendance, with no excessive costs incurred.^99^ However, a recent review suggests a lack of effect compared with usual care.^98^ A large RCT finds no benefit on psychological outcomes or QoL.^100^

### Don’t know

#### Short acting beta agonists (SABA)

There is some evidence for a beneficial effect of albuterol with side effects reported related to heart rate in non COPD contexts.^101–103^ The potential benefit for severe COPD needs careful consideration and high quality evidence is urgently needed.

#### Routine use of ICS

Inconsistent recommendation is made for use of ICS^2,56^ with conflicting evidence. A Cochrane review with good quality evidence suggests routine use of ICS reduces mean rate of exacerbations with no benefits on FEV1 and mortality. Although there is a slower decline in QoL, likelihood of oropharyngeal candidiasis, hoarseness and pneumonia increases.^104^ A possible higher mortality rate is reported in a trial in patients with fluticasone propionate alone compared with placebo.^105^ Research on long term adverse effects is needed.

#### Antidepressants

Short term nortriptyline has some effect on depressive symptoms but not on dyspnoea, FEV1, hospital utilisation, exercise tolerance or QoL. Evidence supporting use of selective serotonin reuptake inhibitors is lacking with reported nausea and dizziness.^106^

#### Benzodiazepines

Benzodiazepines do not improve breathlessness in patients with advanced cancer and COPD regardless of type of benzodiazepine, dose, delivery route and frequency or duration with more adverse events of drowsiness compared with placebo but less compared to morphine.^107^ A longitudinal cohort study observes an association between higher benzodiazepines doses and increased mortality.^108^

#### Routine use of mucolytics

Mucolytics may reduce likelihood of an acute exacerbation. A possible benefit on days of disability and hospitalisations is observed with no association with increased adverse events.^109^

#### Relaxation

Although relaxation is recommended as a way to strengthen coping and functional ability however no high-quality evidence is available to support its routine practice.^110^ A meta-analysis with a high degree of heterogeneity in intervention components concludes only small effects on FEV1, anxiety and QoL.^111^ An explorative RCT suggests benefit of a brief relaxation exercise.^112^ RCT’s evidence is needed to determine comparative effectiveness for this low risk and inexpensive intervention.

#### Breathing exercises

No consistent effects of breathing exercises (with or without supervision) can be drawn on patients’ dyspnoea or QoL,^113^ from a Cochrane review with low-quality evidence. Another review suggests no improvement in dyspnoea but a beneficial effect on respiratory rate.^114^ Further evidence is needed due to large variability among studies examined in current reviews.^115^

## Limitations

A scoping review rather than a systematic review was undertaken. No quality appraisal was performed on supporting evidence. Not all evidence focussed solely on patients with severe COPD. Concerns were given to the current validity of guidelines, all guidelines published within the last 5 years and more than half published within the last 3 years, which is within the timeframe that they may be considered current.^116^

## Summary

A multidisciplinary approach with holistic assessment should be implemented. Treatment with LABA or LAMA is effective, but combination inhaled therapies provide greater benefits without additional adverse effects. However an ICS as part of combination therapy needs to be used with caution considering risk of pneumonia.

- For stable patients, LABA/LAMA and pulmonary rehabilitation are recommended, low dose opioids, self-management, handheld fan and nutritional support may be beneficial. However routine corticosteroids should be avoided.
- For COPD exacerbations, systematic corticosteroids, non-invasive ventilation and exacerbation action plans are recommended, short-acting inhaled beta-agonists and antibiotics may be beneficial but pulmonary rehabilitation should be avoided during hospitalisation.
- Long term oxygen therapy is only recommended for patients with chronic severe hypoxaemia.
- Short-acting anticholinergic inhalers, nebulised opioids, oral theophylline or telehealth are not recommended.

Recommendations by guidelines are not always consistent with robust evidence. There is a lack of high-quality studies focussing on patients with severe COPD. More research is needed to investigate efficacy and safety of ICS, antidepressants, benzodiazepines, mucolytics and supportive care targetting severe COPD.

## Supplemental Material

sj-pdf-1-pmj-10.1177_02692163221079697 – Supplemental material for Evidence-based management approaches for patients with severe chronic obstructive pulmonary disease (COPD): A practice review Click here for additional data file. Supplemental material, sj-pdf-1-pmj-10.1177_02692163221079697 for Evidence-based management approaches for patients with severe chronic obstructive pulmonary disease (COPD): A practice review by Yu Fu, Emma J Chapman, Alison C Boland and Michael I Bennett in Palliative Medicine

## References

[1] Roth GA Abate D Abate KH , et al. Global, regional, and national age-sex-specific mortality for 282 causes of death in 195 countries and territories, 1980–2017: a systematic analysis for the Global Burden of Disease Study 2017. Lancet 2018; 392: 1736–1788. 30496103 10.1016/S0140-6736(18)32203-7 PMC6227606

[2] Global Initiative for Chronic Obstructive Lung Disease (GOLD). Global strategy for the diagnosis, management, and prevention of chronic obstructive pulmonary disease 2020 report. 2020. https://goldcopd.org/wp-content/uploads/2019/12/GOLD-2020-FINAL-ver1.2-03Dec19_WMV.pdf

[3] Halpin DM Miravitlles M Metzdorf N , et al. Impact and prevention of severe exacerbations of COPD: a review of the evidence. Int J Chron Obstruct Pulmon Dis 2017; 12: 2891–2908. 29062228 10.2147/COPD.S139470 PMC5638577

[4] Fu Y Mason A Boland AC , et al. Palliative care needs and integration of palliative care support in COPD: a qualitative study. Chest 2021; 159: 2222–2232. 33434498 10.1016/j.chest.2020.12.055

[5] Bloom CI Slaich B Morales DR , et al. Low uptake of palliative care for COPD patients within primary care in the UK. Eur Respir J 2018; 51: 1701879. 29444916 10.1183/13993003.01879-2017 PMC5898942

[6] Maddocks M Lovell N Booth S , et al. Palliative care and management of troublesome symptoms for people with chronic obstructive pulmonary disease. Lancet 2017; 390: 988–1002. 28872031 10.1016/S0140-6736(17)32127-X

[7] Vermylen JH Szmuilowicz E Kalhan R . Palliative care in COPD: an unmet area for quality improvement. Int J Chron Obstruct Pulmon Dis 2015; 10: 1543–1551. 26345486 10.2147/COPD.S74641 PMC4531041

[8] Tricco AC Lillie E Zarin W , et al. PRISMA extension for scoping reviews (PRISMA-ScR): checklist and explanation. Ann Intern Med 2018; 169: 467–473. 30178033 10.7326/M18-0850

[9] Kruis AL Smidt N Assendelft WJ , et al. Integrated disease management interventions for patients with chronic obstructive pulmonary disease. Cochrane Database Syst Rev 2013; Oct10(10): CD009437. 10.1002/14651858.CD009437.pub2 24108523

[10] Brighton LJ Miller S Farquhar M , et al. Holistic services for people with advanced disease and chronic breathlessness: a systematic review and meta-analysis. Thorax 2019; 74: 270–281. 30498004 10.1136/thoraxjnl-2018-211589 PMC6467249

[11] Higginson IJ Bausewein C Reilly CC , et al. An integrated palliative and respiratory care service for patients with advanced disease and refractory breathlessness: a randomised controlled trial. Lancet Respir Med 2014; 2: 979–987. 25465642 10.1016/S2213-2600(14)70226-7

[12] Cheng S-L Lin C-H Wang C-C , et al. Comparison between COPD Assessment Test (CAT) and modified Medical Research Council (mMRC) dyspnea scores for evaluation of clinical symptoms, comorbidities and medical resources utilization in COPD patients. J Formos Med Assoc 2019; 118: 429–435. 30150099 10.1016/j.jfma.2018.06.018

[13] Çolak Y Nordestgaard BG Vestbo J , et al. Prognostic significance of chronic respiratory symptoms in individuals with normal spirometry. Eur Respir J 2019; 54: 1900734. 31248954 10.1183/13993003.00734-2019

[14] Huang L-H Chen Y-J . The 6-minute walk test to assess exercise capacity of patients with chronic obstructive pulmonary disease. Eur Respir J 2016; 48: PA1385.

[15] Chandra D Wise RA Kulkarni HS , et al. Optimizing the 6-min walk test as a measure of exercise capacity in COPD. Chest 2012; 142: 1545–1552. 23364913 10.1378/chest.11-2702 PMC3515028

[16] Amalakanti S Pentakota MR . Pulse oximetry overestimates oxygen saturation in COPD. Respir Care 2016; 61(4): 423–427. 26715772 10.4187/respcare.04435

[17] Seemungal TA Donaldson GC Paul EA , et al. Effect of exacerbation on quality of life in patients with chronic obstructive pulmonary disease. Am J Respir Crit Care Med 1998; 157: 1418–1422. 9603117 10.1164/ajrccm.157.5.9709032

[18] Schmidt SA Johansen MB Olsen M , et al. The impact of exacerbation frequency on mortality following acute exacerbations of COPD: a registry-based cohort study. BMJ Open 2014; 4: e006720. 10.1136/bmjopen-2014-006720 PMC4275660 25526796

[19] Chen W Thomas J Sadatsafavi M , et al. Risk of cardiovascular comorbidity in patients with chronic obstructive pulmonary disease: a systematic review and meta-analysis. Lancet Respir Med 2015; 3: 631–639. 26208998 10.1016/S2213-2600(15)00241-6

[20] Edgar RG Patel M Bayliss S , et al. Treatment of lung disease in alpha-1 antitrypsin deficiency: a systematic review. Int J Chron Obstruct Pulmon Dis 2017; 12: 1295–1308. 28496314 10.2147/COPD.S130440 PMC5422329

[21] Watz H Tetzlaff K Wouters EF , et al. Blood eosinophil count and exacerbations in severe chronic obstructive pulmonary disease after withdrawal of inhaled corticosteroids: a post-hoc analysis of the WISDOM trial. Lancet Respir Med 2016; 4: 390–398. 27066739 10.1016/S2213-2600(16)00100-4

[22] Geake JB Dabscheck EJ Wood-Baker R , et al. Indacaterol, a once-daily beta2-agonist, versus twice-daily beta2-agonists or placebo for chronic obstructive pulmonary disease. Cochrane Database Syst Rev 2015; 1: CD010139. 25575340 10.1002/14651858.CD010139.pub2 PMC6464646

[23] Kew KM Mavergames C Walters JA . Long-acting beta2-agonists for chronic obstructive pulmonary disease. Cochrane Database Syst Rev 2013; 10: CD010177. 10.1002/14651858.CD010177.pub2 PMC11663505 24127118

[24] Wedzicha JA Calverley PMA Albert RK , et al. Prevention of COPD exacerbations: a European Respiratory Society/American Thoracic Society guideline. Eur Respir J 2017; 50: 1602265. 10.1183/13993003.02265-2016 28889106

[25] Karner C Chong J Poole P . Tiotropium versus placebo for chronic obstructive pulmonary disease. Cochrane Database of Systematic Reviews 2014, Issue 7. Art. No.: CD009285. Epub ahead of print 22 February 2022. DOI: 10.1002/14651858.CD009285.pub3. PMC8934583 25046211

[26] Cheyne L Irvin-Sellers MJ White J . Tiotropium versus ipratropium bromide for chronic obstructive pulmonary disease. Cochrane Database of Systematic Reviews 2015, Issue 9. Art. No.: CD009552. Epub ahead of print 22 February 2022. DOI:10.1002/14651858.CD009552.pub3. PMC8749963 26391969

[27] Koarai A Sugiura H Yamada M , et al. Treatment with LABA versus LAMA for stable COPD: a systematic review and meta-analysis. BMC Pulm Med 2020; 20(1): 111. 32349720 10.1186/s12890-020-1152-8 PMC7191827

[28] NICE. Chronic obstructive pulmonary disease in over 16s: diagnosis and management (NG115). National Institute for Health and Clinical Excellence, London, 2018. 31211541

[29] All Wales Medicines Strategy Group. All Wales COPD management and prescribing guideline. All Wales Therapeutics and Toxicology Centre: Respiratory Health Implementation Group, Penarth, 2020.

[30] Nici L Mammen MJ Charbek E , et al. Pharmacologic management of chronic obstructive pulmonary disease. An official American Thoracic Society clinical practice guideline. Am J Respir Crit Care Med 2020; 201: e56–e69. 32283960 10.1164/rccm.202003-0625ST PMC7193862

[31] Bourbeau Jean Bhutani Mohit Hernandez Paul Aaron Shawn D. Balter Meyer Beauchesne Marie-France D’Urzo Anthony Goldstein Roger Kaplan Alan Maltais François Sin Don D. Marciniuk Darcy D. (2019): Canadian Thoracic Society Clinical Practice Guideline on pharmacotherapy in patients with COPD – 2019 update of evidence, Canadian Journal of Respiratory, Critical Care, and Sleep Medicine, DOI: 10.1080/24745332.2019.1668652

[32] Miravitlles M Soler-Cataluña JJ Calle M , et al. Spanish COPD guidelines (GesEPOC) 2017. Pharmacological treatment of stable chronic obstructive pulmonary disease. Arch Bronconeumol 2017; 53: 324–335. 28477954 10.1016/j.arbres.2017.03.018

[33] D’Urzo A Rennard S Kerwin E , et al. A randomised double-blind, placebo-controlled, long-term extension study of the efficacy, safety and tolerability of fixed-dose combinations of aclidinium/formoterol or monotherapy in the treatment of chronic obstructive pulmonary disease. Respir Med 2017; 125: 39–48. 28340861 10.1016/j.rmed.2017.02.008

[34] Donohue JF Soong W Wu X , et al. Long-term safety of aclidinium bromide/formoterol fumarate fixed-dose combination: results of a randomized 1-year trial in patients with COPD. Respir Med 2016; 116: 41–48. 27296819 10.1016/j.rmed.2016.05.007

[35] Ferguson GT Taylor AF Thach C , et al. Long-term maintenance bronchodilation with indacaterol/glycopyrrolate versus indacaterol in moderate-to-severe COPD patients: the FLIGHT 3 study. Chronic Obstr Pulm Dis 2016; 3: 716–728. 28848898 10.15326/jcopdf.3.4.2016.0131 PMC5556955

[36] Decramer M Anzueto A Kerwin E , et al. Efficacy and safety of umeclidinium plus vilanterol versus tiotropium, vilanterol, or umeclidinium monotherapies over 24 weeks in patients with chronic obstructive pulmonary disease: results from two multicentre, blinded, randomised controlled trials. Lancet Respir Med 2014; 2: 472–486. 24835833 10.1016/S2213-2600(14)70065-7

[37] Farne HA Cates CJ . Long–acting beta2–agonist in addition to tiotropium versus either tiotropium or long–acting beta2–agonist alone for chronic obstructive pulmonary disease. Cochrane Database of Systematic Reviews 2015, Issue 10. Art. No.: CD008989. Epub ahead of print 22 February 2022. DOI: 10.1002/14651858.CD008989. PMC12047630 26490945

[38] Oba Y Keeney E Ghatehorde N , et al. Dual combination therapy versus long-acting bronchodilators alone for chronic obstructive pulmonary disease (COPD): a systematic review and network meta-analysis. Cochrane Database Syst Rev 2018; 12: CD012620. 30521694 10.1002/14651858.CD012620.pub2 PMC6517098

[39] van Geffen WH Carpaij OA Westbroek LF , et al. Long-acting dual bronchodilator therapy (indacaterol/glycopyrronium) versus nebulized short-acting dual bronchodilator (salbutamol/ipratropium) in chronic obstructive pulmonary disease: a double-blind, randomized, placebo-controlled trial. Respir Med 2020; 171: 106064. 32917359 10.1016/j.rmed.2020.106064

[40] Nannini LJ Poole P Milan SJ Kesterton A . Combined corticosteroid and long–acting beta2–agonist in one inhaler versus inhaled corticosteroids alone for chronic obstructive pulmonary disease. Cochrane Database of Systematic Reviews 2013, Issue 8. Art. No.: CD006826. Epub ahead of print 22 February 2022. DOI: 10.1002/14651858.CD006826.pub2. PMC6486274 23990350

[41] Nannini LJ Lasserson TJ Poole P . Combined corticosteroid and long-acting beta2-agonist in one inhaler versus long-acting beta2-agonists for chronic obstructive pulmonary disease. Cochrane Database of Systematic Reviews 2012, Issue 9. Art. No.: CD006829. Epub ahead of print 22 February 2022. DOI: 10.1002/14651858.CD006829.pub2.

[42] Vestbo J Leather D Diar Bakerly N , et al. Effectiveness of fluticasone furoate–vilanterol for COPD in clinical practice. New Engl J Med 2016; 375: 1253–1260. 27593504 10.1056/NEJMoa1608033

[43] Papi A Vestbo J Fabbri L , et al. Extrafine inhaled triple therapy versus dual bronchodilator therapy in chronic obstructive pulmonary disease (TRIBUTE): a double-blind, parallel group, randomised controlled trial. Lancet 2018; 391: 1076–1084. 29429593 10.1016/S0140-6736(18)30206-X

[44] Ferguson GT Rabe KF Martinez FJ , et al. Triple therapy with budesonide/glycopyrrolate/formoterol fumarate with co-suspension delivery technology versus dual therapies in chronic obstructive pulmonary disease (Kronos): a double-blind, parallel-group, multicentre, phase 3 randomised controlled trial. Lancet Respir Med 2018; 6: 747–758. 30232048 10.1016/S2213-2600(18)30327-8

[45] Lipson DA Barnhart F Brealey N , et al. Once-daily single-inhaler triple versus dual therapy in patients with COPD. New Engl J Med 2018; 378: 1671–1680. 29668352 10.1056/NEJMoa1713901

[46] Rojas-Reyes MX García Morales OM Dennis RJ Karner C . Combination inhaled steroid and long-acting beta2-agonist in addition to tiotropium versus tiotropium or combination alone for chronic obstructive pulmonary disease. Cochrane Database of Systematic Reviews 2016, Issue 6. Art. No.: CD008532. Epub ahead of print 22 February 2022 .DOI: 10.1002/14651858.CD008532.pub3. PMC6481546 27271056

[47] Walters JAE Tan DJ White CJ Gibson PG Wood-Baker R Walters EH . Systemic corticosteroids for acute exacerbations of chronic obstructive pulmonary disease. Cochrane Database of Systematic Reviews 2014, Issue 9. Art. No.: CD001288. Epub ahead of print 22 February 2022. DOI: 10.1002/14651858.CD001288.pub4. PMC11195634 25178099

[48] Sivapalan P Ingebrigtsen TS Rasmussen DB , et al. COPD exacerbations: the impact of long versus short courses of oral corticosteroids on mortality and pneumonia: nationwide data on 67 000 patients with COPD followed for 12 months. BMJ Open Respir Res 2019; 6: e000407. 10.1136/bmjresp-2019-000407 PMC6530506 31179005

[49] Johnson MJ Currow DC . Opioids for breathlessness: a narrative review. BMJ Support Palliat Care 2020; 10: 287–295. 10.1136/bmjspcare-2020-002314 32620683

[50] Ekström M Nilsson F Abernethy AA , et al. Effects of opioids on breathlessness and exercise capacity in chronic obstructive pulmonary disease. A systematic review. Ann Am Thorac Soc 2015; 12: 1079–1092. 25803110 10.1513/AnnalsATS.201501-034OC

[51] Verberkt CA van den Beuken-van Everdingen MHJ Schols JMGA , et al. Effect of sustained-release morphine for refractory breathlessness in chronic obstructive pulmonary disease on health status: a randomized clinical trial. JAMA Intern Med 2020; 180: 1306–1314. 32804188 10.1001/jamainternmed.2020.3134 PMC7432282

[52] Currow D Louw S McCloud P , et al. Regular, sustained-release morphine for chronic breathlessness: a multicentre, double-blind, randomised, placebo-controlled trial. Thorax 2020; 75: 50–56. 31558624 10.1136/thoraxjnl-2019-213681

[53] NICE. Chronic obstructive pulmonary disease (acute exacerbation): antimicrobial prescribing. London: National Institute for Health and Care Excellence (NICE), 2018.

[54] Smith D Du Rand I Addy CL , et al. British Thoracic Society guideline for the use of long-term macrolides in adults with respiratory disease. Thorax 2020; 75: 370–404. 32303621 10.1136/thoraxjnl-2019-213929

[55] Wedzicha JA Miravitlles M Hurst JR , et al. Management of COPD exacerbations: a European Respiratory Society/American Thoracic Society guideline. Eur Respir J 2017; 49: 1600791. 10.1183/13993003.00791-2016 28298398

[56] Brown H Bruhl E Bryant K , et al. Diagnosis and Management of Chronic Obstructive Pulmonary Disease (COPD). 2016. https://www.icsi.org/wp-content/uploads/2019/01/COPD.pdf

[57] NICE. COVID-19 rapid guideline: community-based care of patients with chronic obstructive pulmonary disease (COPD). London: National Institute for Clinical Excellence, 2020. 33439588

[58] Uzun S Djamin RS Kluytmans JA , et al. Azithromycin maintenance treatment in patients with frequent exacerbations of chronic obstructive pulmonary disease (COLUMBUS): a randomised, double-blind, placebo-controlled trial. Lancet Respir Med 2014; 2: 361–368. 24746000 10.1016/S2213-2600(14)70019-0

[59] Herath SC Normansell R Maisey S , et al. Prophylactic antibiotic therapy for chronic obstructive pulmonary disease (COPD). Cochrane Database Syst Rev 2018; 10: CD009764. 30376188 10.1002/14651858.CD009764.pub3 PMC6517028

[60] Albert RK Connett J Bailey WC , et al. Azithromycin for prevention of exacerbations of COPD. New Engl J Med 2011; 365: 689–698. 21864166 10.1056/NEJMoa1104623 PMC3220999

[61] Jacobs SS Krishnan JA Lederer DJ , et al. Home oxygen therapy for adults with chronic lung disease. An official American Thoracic Society clinical practice guideline. Am J Respir Crit Care Med 2020; 202: e121–e141. 33185464 10.1164/rccm.202009-3608ST PMC7667898

[62] Gulbas G Gunen H In E , et al. Long-term follow-up of chronic obstructive pulmonary disease patients on long-term oxygen treatment. Int J Clin Pract 2012; 66(2): 152–157. 22188416 10.1111/j.1742-1241.2011.02833.x

[63] Ekström M Ahmadi Z Bornefalk-Hermansson A Abernethy A Currow D . Oxygen for breathlessness in patients with chronic obstructive pulmonary disease who do not qualify for home oxygen therapy. Cochrane Database of Systematic Reviews 2016, Issue 11. Art. No.: CD006429. Epub ahead of print 22 February 2022. DOI: 10.1002/14651858.CD006429.pub3. PMC6464154 27886372

[64] Turner AM Sen S Steeley C , et al. Evaluation of oxygen prescription in relation to hospital admission rate in patients with chronic obstructive pulmonary disease. BMC Pulm Med 2014; 14(1): 127. 25096821 10.1186/1471-2466-14-127 PMC4129429

[65] Hardinge M Suntharalingam J Wilkinson T . Guideline update: The British Thoracic Society Guidelines on home oxygen use in adults. Thorax 2015; 70: 589–591. 25918120 10.1136/thoraxjnl-2015-206918

[66] Abernethy AP McDonald CF Frith PA , et al. Effect of palliative oxygen versus room air in relief of breathlessness in patients with refractory dyspnoea: a double-blind, randomised controlled trial. Lancet 2010; 376: 784–793. 20816546 10.1016/S0140-6736(10)61115-4 PMC2962424

[67] Chandra D Stamm JA Taylor B , et al. Outcomes of noninvasive ventilation for acute exacerbations of chronic obstructive pulmonary disease in the United States, 1998-2008. Am J Respir Crit Care Med 2012; 185: 152–159. 22016446 10.1164/rccm.201106-1094OC PMC3297087

[68] Osadnik CR Tee VS Carson-Chahhoud KV , et al. Non-invasive ventilation for the management of acute hypercapnic respiratory failure due to exacerbation of chronic obstructive pulmonary disease.. Cochrane Database Syst Rev 2017; 7: CD004104. 28702957 10.1002/14651858.CD004104.pub4 PMC6483555

[69] Dretzke J Moore D Dave C , et al. The effect of domiciliary noninvasive ventilation on clinical outcomes in stable and recently hospitalized patients with COPD: a systematic review and meta-analysis. Int J Chron Obstruct Pulmon Dis 2016; 11: 2269–2286. 27698560 10.2147/COPD.S104238 PMC5034919

[70] Bhatt SP Peterson MW Wilson JS , et al. Noninvasive positive pressure ventilation in subjects with stable COPD: a randomized trial. Int J Chron Obstruct Pulmon Dis 2013; 8: 581–589. 24293994 10.2147/COPD.S53619 PMC3842217

[71] Zwerink M Brusse-Keizer M van der Valk PDLPM Zielhuis GA Monninkhof EM van der Palen J Frith PA Effing T . Self management for patients with chronic obstructive pulmonary disease. Cochrane Database of Systematic Reviews 2014, Issue 3. Art. No.: CD002990. Epub ahead of print 22 February 2022. DOI: 10.1002/14651858.CD002990.pub3. PMC7004246 24665053

[72] Zwerink M Brusse-Keizer M van der Valk PD , et al. Self management for patients with chronic obstructive pulmonary disease. Cochrane Database Syst Rev 2014; 3: CD002990. 10.1002/14651858.CD002990.pub3 PMC7004246 24665053

[73] Lenferink A Brusse-Keizer M van der Valk PD , et al. Self-management interventions including action plans for exacerbations versus usual care in patients with chronic obstructive pulmonary disease. Cochrane Database Syst Rev 2017; 8: CD011682. 28777450 10.1002/14651858.CD011682.pub2 PMC6483374

[74] Howcroft M Walters EH Wood-Baker R , et al. Action plans with brief patient education for exacerbations in chronic obstructive pulmonary disease. Cochrane Database Syst Rev 2016; 12: CD005074. 27990628 10.1002/14651858.CD005074.pub4 PMC6463844

[75] McCarthy B Casey D Devane D Murphy K Murphy E Lacasse Y . Pulmonary rehabilitation for chronic obstructive pulmonary disease. Cochrane Database of Systematic Reviews 2015, Issue 2. Art. No.: CD003793. Epub ahead of print 22 February 2022. DOI: 10.1002/14651858.CD003793.pub3. PMC10008021 25705944

[76] Puhan MA Gimeno-Santos E Cates CJ , et al. Pulmonary rehabilitation following exacerbations of chronic obstructive pulmonary disease. Cochrane Database Syst Rev 2016; 12: CD005305. 27930803 10.1002/14651858.CD005305.pub4 PMC6463852

[77] Holland AE Mahal A Hill CJ , et al. Home-based rehabilitation for COPD using minimal resources: a randomised, controlled equivalence trial. Thorax 2017; 72: 57–65. 27672116 10.1136/thoraxjnl-2016-208514 PMC5329049

[78] Güell M-R Cejudo P Ortega F , et al. Benefits of long-term pulmonary rehabilitation maintenance program in patients with severe chronic obstructive pulmonary disease. three-year follow-up. Am J Respir Crit Care Med 2017; 195: 622–629. 27611807 10.1164/rccm.201603-0602OC

[79] Pollok J van Agteren JE Esterman AJ , et al. Psychological therapies for the treatment of depression in chronic obstructive pulmonary disease. Cochrane Database Syst Rev 2019; 3: CD012347. 30838649 10.1002/14651858.CD012347.pub2 PMC6400788

[80] Zhang X Yin C Tian W , et al. Effects of cognitive behavioral therapy on anxiety and depression in patients with chronic obstructive pulmonary disease: a meta-analysis and systematic review. Clin Respir J 2020; 14: 891–900. 32510764 10.1111/crj.13226

[81] Baraniak A Sheffield D . The efficacy of psychologically based interventions to improve anxiety, depression and quality of life in COPD: a systematic review and meta-analysis. Patient Educ Couns 2011; 83: 29–36. 20447795 10.1016/j.pec.2010.04.010

[82] Heslop-Marshall K Baker C Carrick-Sen D , et al. Randomised controlled trial of cognitive behavioural therapy in COPD. ERJ Open Res 2018; 4: 00094–02018. 10.1183/23120541.00094-2018 PMC6250562 30479999

[83] Coventry PA Bower P Keyworth C , et al. The effect of complex interventions on depression and anxiety in chronic obstructive pulmonary disease: systematic review and meta-analysis. PLoS One 2013; 8: e60532. 23585837 10.1371/journal.pone.0060532 PMC3621386

[84] Johnson MJ Booth S Currow DC , et al. A mixed-methods, randomized, controlled feasibility trial to inform the design of a phase III trial to test the effect of the handheld fan on physical activity and carer anxiety in patients with refractory breathlessness. J Pain Symptom Manag 2016; 51: 807–815. 10.1016/j.jpainsymman.2015.11.026 26880253

[85] Barnes-Harris M Allgar V Booth S , et al. Battery operated fan and chronic breathlessness: does it help? BMJ Support Palliat Care 2019; 9: 478–481. 10.1136/bmjspcare-2018-001749 31068332

[86] Galbraith S Fagan P Perkins P , et al. Does the use of a handheld fan improve chronic dyspnea? A randomized, controlled, crossover trial. J Pain Symptom Manag 2010; 39: 831–838. 10.1016/j.jpainsymman.2009.09.024 20471544

[87] Marchetti N Lammi MR Travaline JM , et al. Air current applied to the face improves exercise performance in patients with COPD. Lung 2015; 193(5): 725–731. 26255060 10.1007/s00408-015-9780-0 PMC4996349

[88] Ferreira IM Brooks D White J , et al. Nutritional supplementation for stable chronic obstructive pulmonary disease. Cochrane Database Syst Rev 2012; 12: CD000998. 23235577 10.1002/14651858.CD000998.pub3 PMC11742366

[89] Collins PF Elia M Stratton RJ . Nutritional support and functional capacity in chronic obstructive pulmonary disease: a systematic review and meta-analysis. Respirology 2013; 18: 616–629. 23432923 10.1111/resp.12070

[90] Jolliffe DA Greenberg L Hooper RL , et al. Vitamin D to prevent exacerbations of COPD: systematic review and meta-analysis of individual participant data from randomised controlled trials. Thorax 2019; 74: 337–345. 30630893 10.1136/thoraxjnl-2018-212092

[91] Walters JA Tan DJ White CJ , et al. Different durations of corticosteroid therapy for exacerbations of chronic obstructive pulmonary disease. Cochrane Database Syst Rev 2018; 3: CD006897. 29553157 10.1002/14651858.CD006897.pub4 PMC6494402

[92] Barnes H McDonald J Smallwood N , et al. Opioids for the palliation of refractory breathlessness in adults with advanced disease and terminal illness. Cochrane Database Syst Rev 2016; 3: CD011008. 27030166 10.1002/14651858.CD011008.pub2 PMC6485401

[93] Wilairat P Kengkla K Thayawiwat C , et al. Clinical outcomes of theophylline use as add-on therapy in patients with chronic obstructive pulmonary disease: a propensity score matching analysis. Chron Respir Dis 2019; 16: 1479973118815694. 30558448 10.1177/1479973118815694 PMC6302972

[94] Jenkins CR Wen F-Q Martin A , et al. The effect of low-dose corticosteroids and theophylline on the risk of acute exacerbations of COPD: the TASCS randomised controlled trial. Eur Respir J 2021; 57: 2003338. 33334939 10.1183/13993003.03338-2020

[95] Janjua S Fortescue R Poole P . Phosphodiesterase-4 inhibitors for chronic obstructive pulmonary disease. Cochrane Database Syst Rev 2020; 5: CD002309. 32356609 10.1002/14651858.CD002309.pub6 PMC7193764

[96] Greening NJ Williams JEA Hussain SF , et al. An early rehabilitation intervention to enhance recovery during hospital admission for an exacerbation of chronic respiratory disease: randomised controlled trial. BMJ 2014; 349: g4315. 25004917 10.1136/bmj.g4315 PMC4086299

[97] Tang CY Blackstock FC Clarence M , et al. Early rehabilitation exercise program for inpatients during an acute exacerbation of chronic obstructive pulmonary disease: a randomized controlled trial. J Cardiopulm Rehabil Prev 2012; 32(3): 163–169. 22561417 10.1097/HCR.0b013e318252f0b2

[98] Gregersen TL Green A Frausing E , et al. Do telemedical interventions improve quality of life in patients with COPD? A systematic review. Int J Chron Obstruct Pulmon Dis 2016; 11: 809–822. 27143872 10.2147/COPD.S96079 PMC4846042

[99] McLean S Nurmatov U Liu JLY Pagliari C Car J Sheikh A . Telehealthcare for chronic obstructive pulmonary disease. Cochrane Database of Systematic Reviews 2011, Issue 7. Art. No.: CD007718. Epub ahead of print 22 February 2022. DOI: 10.1002/14651858.CD007718.pub2. PMC8939044 21735417

[100] Cartwright M Hirani SP Rixon L , et al. Effect of telehealth on quality of life and psychological outcomes over 12 months (Whole Systems Demonstrator telehealth questionnaire study): nested study of patient reported outcomes in a pragmatic, cluster randomised controlled trial. BMJ 2013; 346: f653. 23444424 10.1136/bmj.f653 PMC3582704

[101] Wilkinson M Bulloch B Garcia-Filion P , et al. Efficacy of racemic albuterol versus levalbuterol used as a continuous nebulization for the treatment of acute asthma exacerbations: a randomized, double-blind, clinical trial. J Asthma 2011; 48: 188–193. 21275850 10.3109/02770903.2011.554939

[102] Jat KR Khairwa A . Levalbuterol versus albuterol for acute asthma: a systematic review and meta-analysis. Pulm Pharmacol Ther 2013; 26(2): 239–248. 23207739 10.1016/j.pupt.2012.11.003

[103] Brunetti L Poiani G Dhanaliwala F , et al. Clinical outcomes and treatment cost comparison of levalbuterol versus albuterol in hospitalized adults with chronic obstructive pulmonary disease or asthma. Am J Health Syst Pharm 2015; 72: 1026–1035. 26025994 10.2146/ajhp140551

[104] Yang IA Clarke MS Sim EHA Fong KM . Inhaled corticosteroids for stable chronic obstructive pulmonary disease. Cochrane Database of Systematic Reviews 2012, Issue 7. Art. No.: CD002991. Epub ahead of print 22 February 2022. DOI: 10.1002/14651858.CD002991.pub3. PMC8992433 22786484

[105] Vestbo J Anderson JA Brook RD , et al. Fluticasone furoate and vilanterol and survival in chronic obstructive pulmonary disease with heightened cardiovascular risk (SUMMIT): a double-blind randomised controlled trial. Lancet 2016; 387: 1817–1826. 27203508 10.1016/S0140-6736(16)30069-1

[106] Pollok J van Agteren JE Carson-Chahhoud KV . Pharmacological interventions for the treatment of depression in chronic obstructive pulmonary disease. Cochrane Database Syst Rev 2018; 12: CD012346. 30566235 10.1002/14651858.CD012346.pub2 PMC6517114

[107] Simon ST Higginson IJ Booth S , et al. Benzodiazepines for the relief of breathlessness in advanced malignant and non-malignant diseases in adults.. Cochrane Database Syst Rev 2016; 10: CD007354. 27764523 10.1002/14651858.CD007354.pub3 PMC6464146

[108] Ekström MP Bornefalk-Hermansson A Abernethy AP , et al. Safety of benzodiazepines and opioids in very severe respiratory disease: national prospective study. BMJ 2014; 348: g445. 24482539 10.1136/bmj.g445 PMC3906915

[109] Poole P Sathananthan K Fortescue R . Mucolytic agents versus placebo for chronic bronchitis or chronic obstructive pulmonary disease. Cochrane Database Syst Rev 2019; 5: CD001287. 31107966 10.1002/14651858.CD001287.pub6 PMC6527426

[110] NHS Scotland. Scottish palliative care guidlines - breathlessness. Scotland: NHS Scotland, Glasgow, 2019.

[111] Volpato E Banfi P Rogers SM , et al. Relaxation techniques for people with chronic obstructive pulmonary disease: a systematic review and a meta-analysis. Evid Based Complement Alternat Med 2015; 2015: 628365. 26339268 10.1155/2015/628365 PMC4539049

[112] Volpato E Banfi P Nicolini A , et al. A quick relaxation exercise for people with chronic obstructive pulmonary disease: explorative randomized controlled trial. Multidiscip Respir Med 2018; 13(1): 13. 29744054 10.1186/s40248-018-0124-9 PMC5932751

[113] Holland AE Hill CJ Jones AY , et al. Breathing exercises for chronic obstructive pulmonary disease. Cochrane Database Syst Rev 2012; 10: CD008250. 23076942 10.1002/14651858.CD008250.pub2 PMC11371308

[114] Ubolnuar N Tantisuwat A Thaveeratitham P , et al. Effects of breathing exercises in patients with chronic obstructive pulmonary disease: systematic review and meta-analysis. Ann Rehabil Med 2019; 43: 509–523. 31499605 10.5535/arm.2019.43.4.509 PMC6734022

[115] Borge CR Hagen KB Mengshoel AM , et al. Effects of controlled breathing exercises and respiratory muscle training in people with chronic obstructive pulmonary disease: results from evaluating the quality of evidence in systematic reviews. BMC Pulm Med 2014; 14(1): 184. 25416306 10.1186/1471-2466-14-184 PMC4258938

[116] Shekelle PG Ortiz E Rhodes S , et al. Validity of the Agency for healthcare research and quality clinical practice guidelines: how quickly do guidelines become outdated? JAMA 2001; 286: 1461–1467. 11572738 10.1001/jama.286.12.1461

