# Supplementary material for: Evidence-based management approaches for patients with severe chronic obstructive pulmonary disease (COPD): A practice review
Source: Palliat Med. 2022 Mar 20;36(5):770–82. doi: 10.1177/02692163221079697 (PMC9087316; doi:10.1177/02692163221079697)
Supplement: sj-pdf-1-pmj-10.1177_02692163221079697 – Supplemental material for Evidence-based management approaches for patients with severe chronic obstructive pulmonary disease (COPD): A practice review [file sj-pdf-1-pmj-10.1177_02692163221079697.pdf]

Supplementary material 1 Search strategies

<u>PubMed search strategy</u>

(Chronic obstructive pulmonary disease or COPD) [Title/Abstract] AND Guideline [Title/Abstract]

(Multidisciplinary or Assessment) [Title/Abstract] AND (Chronic obstructive pulmonary disease or COPD) [Title/Abstract]

(Beta-agonist or Anticholinergics) [Title/Abstract] AND (Chronic obstructive pulmonary disease or COPD) [Title/Abstract]

(Inhaled corticosteroids or Corticosteroids or Opioids or Antibiotics or Theophylline or Phosphodiesterase-4 or Antidepressants or Benzodiazepines or Mucolytics) [Title/Abstract] AND (Chronic obstructive pulmonary disease or COPD) [Title/Abstract]

(Oxygen therapy or Non-invasive ventilation or Self-management or Pulmonary rehabilitation or Cognitive Behavioural Therapy or Handheld fan or Nutritional support or Telehealth or Relaxation or Breathing exercise) [Title/Abstract] AND (Chronic obstructive pulmonary disease or COPD) [Title/Abstract]

Supplementary material 2 Selection process of guidelines_PRISMA flowchart

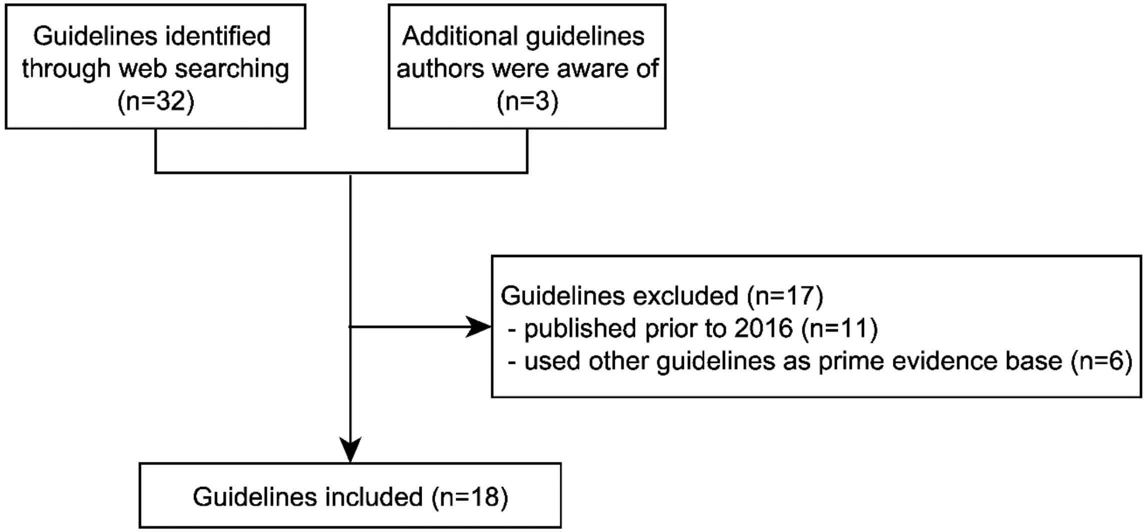

Supplementary material 3 Evidence used to form practice recommendations

Authors	Year	Title	Source	Type of evidence
Do_ multidisciplinary approach and assessment				
Kruis AL, Smidt N, Assendelft WJ, Gussekloo J, Boland MR, Rutten‐van Mölken M, Chavannes NH	2013	Integrated disease management interventions for patients with chronic obstructive pulmonary disease	Cochrane Database of Systematic Reviews, 2013 (10)	Cochrane review
Brighton LJ, Miller S, Farquhar M, Booth S, Yi D, Gao W, Bajwah S, Man WD, Higginson IJ, Maddocks M	2019	Holistic services for people with advanced disease and chronic breathlessness: a systematic review and meta-analysis	Thorax 2019: 74(3): 270-281.	Systematic review and meta-analysis
Higginson IJ, Bausewein C, Reilly CC, Gao W, Gysels M, Dzingina M, McCrone P, Booth S, Jolley CJ, Moxham J.	2014	An integrated palliative and respiratory care service for patients with advanced disease and refractory breathlessness: a randomised controlled trial	The Lancet Respiratory Medicine 2014: 2(12): 979-987	Randomised controlled trial
Çolak Y, Nordestgaard BG, Vestbo J, Lange P, Afzal S.	2019	Prognostic significance of chronic respiratory symptoms in individuals with normal spirometry	European Respiratory Journal 2019: 54(3): 1900734	Population-based cohort study
Cheng S-L, Lin C-H, Wang C-C, Chan M-C, Hsu J-Y, Hang L-W, Perng D-W, Yu C-J, Wang H-C.	2019	Comparison between COPD Assessment Test (CAT) and modified Medical Research Council (mMRC) dyspnea scores for evaluation of clinical symptoms, comorbidities and medical resources utilization in COPD patients	Journal of the Formosan Medical Association 2019: 118(1, Part 3): 429-435	Population based cross-sectional study
Huang L-H, Chen Y-J.	2016	The 6-minute walk test to assess exercise capacity of patients with chronic obstructive pulmonary disease	European Respiratory Journal 2016: 48(suppl 60): PA1385	Systematic review
Chandra D, Wise RA, Kulkarni HS, Benzo RP, Criner G, Make B, Slivka WA, Ries AL, Reilly JJ, Martinez FJ, Sciurba FC	2012	Optimizing the 6-Min Walk Test as a Measure of Exercise Capacity in COPD	CHEST 2012: 142(6): 1545-1552	Cohort study
Amalakanti S, Pentakota MR.	2016	Pulse Oximetry Overestimates Oxygen Saturation in COPD	Respir Care 2016: 61(4): 423-427.	Cross-sectional observational study
Schmidt SAJ, Johansen MB, Olsen M, Xu X, Parker JM, Molfino NA, Lash TL, Sørensen HT, Christiansen CF.	2014	The impact of exacerbation frequency on mortality following acute exacerbations of COPD: a registry-based cohort study	BMJ Open 214: 4(12): e006720	Registry-based cohort study

Chen W, Thomas J, Sadatsafavi M, FitzGerald JM.		2015	Risk of cardiovascular comorbidity in patients with chronic obstructive pulmonary disease: a systematic review and meta-analysis	The Lancet Respiratory Medicine 2015: 3(8): 631-639	Systematic review and meta-analysis
Edgar RG, Patel M, Bayliss S, Crossley D, Sapey E, Turner AM.		2017	Treatment of lung disease in alpha-1 antitrypsin deficiency: a systematic review	International journal of chronic obstructive pulmonary disease 2017: 12: 1295-1308	Systematic review
Watz H,Tetzlaff K, Wouters E F. M, Kirsten A, Magnussen H, Rodriguez-Roisin R, Vogelmeier C, Fabbri L M, Chanez P, Dahl R, Disse B, Finnigan H, Calverley P. M. A.		2016	Blood eosinophil count and exacerbations in severe chronic obstructive pulmonary disease after withdrawal of inhaled corticosteroids: a post-hoc analysis of the WISDOM trial.	The Lancet Respiratory Medicine. 2016; 4: 390-8.	Randomized, controlled study
Do_ pharmacological management					
Beta-agonist	Geake JB, Dabscheck EJ, Wood - Baker R, Cates CJ.	2015	Indacaterol, a once - daily beta2 - agonist, versus twice - daily beta2 - agonists or placebo for chronic obstructive pulmonary disease.	Cochrane Database of Systematic Reviews, 2015(1).	Cochrane review
	Kew KM, Mavergames C, Walters JAE.	2013	Long - acting beta2 - agonists for chronic obstructive pulmonary disease.	Cochrane Database of Systematic Reviews, 2013 (10)	Cochrane review
Anticholinergics	Karner C, Chong J, Poole P.	2014	Tiotropium versus placebo for chronic obstructive pulmonary disease.	Cochrane Database of Systematic Reviews 2014(7).	Cochrane review
	Cheyne L, Irvin - Sellers MJ, White J.	2015	Tiotropium versus ipratropium bromide for chronic obstructive pulmonary disease.	Cochrane Database of Systematic Reviews 2015(9).	Cochrane review
	Koarai A, Sugiura H, Yamada M, Ichikawa T, Fujino N, Kawayama T, Ichinose M.	2020	Treatment with LABA versus LAMA for stable COPD: a systematic review and meta-analysis.	BMC Pulmonary Medicine 2020: 20(1): 111	Systematic review and meta-analysis
SABA/SAMA	Ferguson GT, Ghafouri M, Dai L, Dunn LJ.	2013	COPD patient satisfaction with ipratropium bromide/albuterol delivered via Respimat: a randomized, controlled study.	International journal of chronic obstructive pulmonary disease 2013: 8: 139-150	Randomized, controlled study

LABA/LAMA	Donohue JF, Soong W, Wu X, Shrestha P and Lei A.		Long-term safety of aclidinium bromide/formoterol fumarate fixed-dose combination: Results of a randomized 1-year trial in patients with COPD.	Respir Med. 2016; 116: 41-8.	Randomized, controlled study
	D'Urzo A, Rennard S, Kerwin E, et al	2017	A randomised double-blind, placebo-controlled, long-term extension study of the efficacy, safety and tolerability of fixed-dose combinations of aclidinium/formoterol or monotherapy in the treatment of chronic obstructive pulmonary disease.	Respiratory Medicine. 2017; 125: 39-48.	Randomized, controlled study
	Ferguson GT, Taylor AF, Thach C, et al.	2016	Long-term maintenance bronchodilation with indacaterol/glycopyrrolate versus indacaterol in moderate-to-severe COPD patients: the FLIGHT 3 study.	Chronic Obstructive Pulmonary Diseases. 2016; 3: 716.	Randomized, controlled study
	Decramer M, Anzueto A, Kerwin E, Kaelin T, Richard N, Crater G, Tabberer M, Harris S, Church A	2014	Efficacy and safety of umeclidinium plus vilanterol versus tiotropium, vilanterol, or umeclidinium monotherapies over 24 weeks in patients with chronic obstructive pulmonary disease: results from two multicentre, blinded, randomised controlled trials.	The Lancet Respiratory Medicine. 2014; 2: 472-86.	Randomized, controlled study
	Farne HA, Cates CJ.	2015	Long‐acting beta2‐agonist in addition to tiotropium versus either tiotropium or long‐acting beta2‐agonist alone for chronic obstructive pulmonary disease.	Cochrane Database of Systematic Reviews 2015(10).	Cochrane review
	Oba Y, Keeney E, Ghatehorde N, Dias S.	2018	Dual combination therapy versus long‐acting bronchodilators alone for chronic obstructive pulmonary disease (COPD): a systematic review and network meta‐analysis.	Cochrane Database of Systematic Reviews 2018(12).	Cochrane review
	van Geffen WH, Carpaij OA, Westbroek LF, Seigers D, Niemeijer A, Vonk JM, Kerstjens HAM..	2020	Long-acting dual bronchodilator therapy (indacaterol/glycopyrronium) versus nebulized short-acting dual bronchodilator (salbutamol/ipratropium) in chronic obstructive pulmonary disease: A double-blind, randomized, placebo-controlled trial	Respiratory Medicine 2020: 171: 106064.	Randomised, controlled study
ICS/LABA	Nannini LJ, Poole P, Milan SJ, Kesterton A.	2013	Combined corticosteroid and long‐acting beta2‐agonist in one inhaler versus inhaled corticosteroids alone for chronic obstructive pulmonary disease.	Cochrane Database of Systematic Reviews 2013(8).	Cochrane review

	Nannini LJ, Lasserson TJ, Poole P.	2012	Combined corticosteroid and long‐acting beta2‐agonist in one inhaler versus long‐acting beta2‐agonists for chronic obstructive pulmonary disease.	Cochrane Database of Systematic Reviews 2012(9).	Cochrane review
	Vestbo J, Leather D, Diar Bakerly N, New J, Gibson JM, McCorkindale S, Collier S, Crawford J, Frith L, Harvey C, Svedsater H, Woodcock A.	2016	Effectiveness of Fluticasone Furoate–Vilanterol for COPD in Clinical Practice.	New England Journal of Medicine 2016: 375(13): 1253-1260	Randomised, controlled study
ICS/LABA/LAMA	Papi, A, Vestbo, J, Fabbri, L. Corradi, M.Prunier, H. Cohuet, G.Guasconi, A. Montagna, I.Vezzoli, S. Petruzzelli, S.Scuri, M. Roche, N.Singh, D.	2018	Papi A, Vestbo J, Fabbri L, et al. Extrafine inhaled triple therapy versus dual bronchodilator therapy in chronic obstructive pulmonary disease (TRIBUTE): a double-blind, parallel group, randomised controlled trial.	Lancet. 2018; 391: 1076-84.	Randomised, controlled study
	Rojas‐Reyes MX, García Morales OM, Dennis RJ, Karner C.	2016	Combination inhaled steroid and long‐acting beta2‐agonist in addition to tiotropium versus tiotropium or combination alone for chronic obstructive pulmonary disease.	Cochrane Database of Systematic Reviews 2016(6).	Cochrane review
	Ferguson GT, Rabe KF, Martinez FJ, Fabbri LM, Wang C, Ichinose M, Bourne E, Ballal S, Darken P, DeAngelis K, Aurivillius M, Dorinsky P, Reisner C.	2018	Triple therapy with budesonide/glycopyrrolate/formoterol fumarate with co-suspension delivery technology versus dual therapies in chronic obstructive pulmonary disease (KRONOS): a double-blind, parallel-group, multicentre, phase 3 randomised controlled trial.	The Lancet Respiratory Medicine 2018: 6(10): 747-758.	Randomised, controlled study
	Lipson DA, Barnhart F, Brealey N, Brooks J, Criner GJ, Day NC, Dransfield MT, Halpin DMG, Han MK, Jones CE, Kilbride S, Lange P, Lomas DA, Martinez FJ,	2018	Once-Daily Single-Inhaler Triple versus Dual Therapy in Patients with COPD.	New England Journal of Medicine 2018: 378(18): 1671-1680.	Randomised, controlled study

	Singh D, Tabberer M, Wise RA, Pascoe SJ.				
Systemic corticosteroids in acute exacerbations	Walters JAE, Tan DJ, White CJ, Gibson PG, Wood‐Baker R, Walters EH.	2014	Systemic corticosteroids for acute exacerbations of chronic obstructive pulmonary disease.	Cochrane Database of Systematic Reviews 2014(9).	Cochrane review
	Sivapalan P, Ingebrigtsen TS, Rasmussen DB, Sørensen R, Rasmussen CM, Jensen CB, Allin KH, Eklöf J, Seersholm N, Vestbo J, Jensen J-US.	2019	COPD exacerbations: the impact of long versus short courses of oral corticosteroids on mortality and pneumonia: nationwide data on 67 000 patients with COPD followed for 12 months	BMJ open respiratory research. 2019; 6: e000407-e	Observational study
Opioids	Johnson MJ, Currow DC.	2020	Opioids for breathlessness: a narrative review.	BMJ Supportive & Palliative Care 2020: 10(3): 287-295.	Narrative review
	Ekström M, Nilsson F, Abernethy AA, Currow DC.	2015	Effects of Opioids on Breathlessness and Exercise Capacity in Chronic Obstructive Pulmonary Disease. A Systematic Review.	Annals of the American Thoracic Society 2015: 12(7): 1079-1092.	Systematic review
	Verberkt CA, van den Beuken-van Everdingen MHJ, Schols JMGA, Hameleers N, Wouters EFM, Janssen DJA.	2020	Effect of Sustained-Release Morphine for Refractory Breathlessness in Chronic Obstructive Pulmonary Disease on Health Status: A Randomized Clinical Trial.	JAMA Internal Medicine 2020: 180(10): 1306-1314.	Randomised, controlled study
	Currow D, Louw S, McCloud P, Fazekas B, Plummer J, McDonald CF, Agar M, Clark K, McCaffery N, Ekström MP.	2020	Regular, sustained-release morphine for chronic breathlessness: a multicentre, double-blind, randomised, placebo-controlled trial.	Thorax 2020: 75(1): 50-56.	Randomised, controlled study
Antibiotics	Uzun S, Djamin RS, Kluytmans JA, Mulder PG, van't Veer NE, Ermens AA, Pelle AJ, Hoogsteden	2014	Azithromycin maintenance treatment in patients with frequent exacerbations of chronic obstructive pulmonary disease (COLUMBUS): a randomised, double-blind, placebo-controlled trial.	Lancet Respir Med 2014: 2(5): 361-368.	Randomised, controlled study

	HC, Aerts JG, van der Eerden MM.				
	Herath SC, Normansell R, Maisey S, Poole P.	2018	Prophylactic antibiotic therapy for chronic obstructive pulmonary disease (COPD).	Cochrane Database of Systematic Reviews 2018(10).	Cochrane review
	Albert RK, Connett J, Bailey WC, Casaburi R, Cooper JAD, Criner GJ, Curtis JL, Dransfield MT, Han MK, Lazarus SC, Make B, Marchetti N, Martinez FJ, Madinger NE, McEvoy C, Niewoehner DE, Porsasz J, Price CS, Reilly J, Scanlon PD, Sciurba FC, Scharf SM, Washko GR, Woodruff PG, Anthonisen NR.	2011	Azithromycin for Prevention of Exacerbations of COPD.	New England Journal of Medicine 2011: 365(8): 689-698.	Randomised, controlled study
Do_LTOT					
Gulbas G, Gunen H, In E, Kilic T.		2012	Long-term follow-up of chronic obstructive pulmonary disease patients on long-term oxygen treatment.	International Journal of Clinical Practice 2012: 66(2): 152-157.	Cohort study
Ekström M, Ahmadi Z, Bornefalk‐Hermansson A, Abernethy A, Currow D.		2016	Oxygen for breathlessness in patients with chronic obstructive pulmonary disease who do not qualify for home oxygen therapy.	Cochrane Database of Systematic Reviews 2016(11).	Systematic review
Turner AM, Sen S, Steeley C, Khan Y, Sweeney P, Richards Y, Mukherjee R.		2014	Evaluation of oxygen prescription in relation to hospital admission rate in patients with chronic obstructive pulmonary disease.	BMC Pulm Med 2014: 14: 127	Cohort study

Abernethy AP, McDonald CF, Frith PA, Clark K, Herndon JE, II, Marcello J, Young IH, Bull J, Wilcock A, Booth S, Wheeler JL, Tulsky JA, Crockett AJ, Currow DC.	2010	Effect of palliative oxygen versus room air in relief of breathlessness in patients with refractory dyspnoea: a double-blind, randomised controlled trial.	The Lancet 2010: 376(9743): 784-793.	Randomised controlled study
Do_NIV				
Osadnik CR, Tee VS, Carson‐Chahhoud KV, Picot J, Wedzicha JA, Smith BJ.	2017	Non‐invasive ventilation for the management of acute hypercapnic respiratory failure due to exacerbation of chronic obstructive pulmonary disease.	Cochrane Database of Systematic Reviews 2017(7).	Cochrane review
Chandra D, Stamm JA, Taylor B, Ramos RM, Satterwhite L, Krishnan JA, Mannino D, Sciurba FC, Holguin F.	212	Outcomes of noninvasive ventilation for acute exacerbations of chronic obstructive pulmonary disease in the United States, 1998-2008.	Am J Respir Crit Care Med. 2012; 185: 152-9	Cohort study
Dretzke J, Moore D, Dave C, Mukherjee R, Price MJ, Bayliss S, Wu X, Jordan RE, Turner AM.	2016	The effect of domiciliary noninvasive ventilation on clinical outcomes in stable and recently hospitalized patients with COPD: a systematic review and meta-analysis.	International journal of chronic obstructive pulmonary disease 2016: 11: 2269-2286.	Systematic review and meta-analysis.
Bhatt SP, Peterson MW, Wilson JS, Durairaj L.	2013	Noninvasive positive pressure ventilation in subjects with stable COPD: a randomized trial.	International journal of chronic obstructive pulmonary disease 2013: 8: 581-589.	Randomised, controlled study
Köhnlein T, Windisch W, Köhler D, Drabik A, Geiseler J, Hartl S, Karg O, Laier-Groeneveld G, Nava S, Schönhofer B, Schucher B, Wegscheider K, Criée CP, Welte T.	2014	Non-invasive positive pressure ventilation for the treatment of severe stable chronic obstructive pulmonary disease: a prospective, multicentre, randomised, controlled clinical trial.	The Lancet Respiratory Medicine 2014: 2(9): 698-705	Randomised, controlled study
Do_self-management (moderate)				
Zwerink M, Brusse‐Keizer M, van der Valk P, Zielhuis GA, Monninkhof EM, van der Palen J, Frith PA, Effing T.	2014	Self management for patients with chronic obstructive pulmonary disease.	Cochrane Database of Systematic Reviews 2014(3).	Cochrane review
Lenferink A, Brusse‐Keizer M, van der Valk P, Frith PA, Zwerink M, Monninkhof EM, van der Palen J, Effing TW.	2017	Self‐management interventions including action plans for exacerbations versus usual care in patients with chronic obstructive pulmonary disease.	Cochrane Database of Systematic Reviews 2017(8).	Cochrane review

Howcroft M, Walters EH, Wood-Baker R and Walters JA.	2016	Action plans with brief patient education for exacerbations in chronic obstructive pulmonary disease.	Cochrane Database of Systematic Reviews. 2016 (12).	Cochrane review
Do_PR				
McCarthy B, Casey D, Devane D, Murphy K, Murphy E, Lacasse Y.	2015	Pulmonary rehabilitation for chronic obstructive pulmonary disease.	Cochrane Database of Systematic Reviews 2015(2)	Cochrane review
Puhan MA, Gimeno - Santos E, Cates CJ, Troosters T.	2016	Pulmonary rehabilitation following exacerbations of chronic obstructive pulmonary disease.	Cochrane Database of Systematic Reviews 2016(12).	Cochrane review
Holland AE, Mahal A, Hill CJ, Lee AL, Burge AT, Cox NS, Moore R, Nicolson C, O'Halloran P, Lahham A, Gillies R, McDonald CF.	2017	Home-based rehabilitation for COPD using minimal resources: a randomised, controlled equivalence trial.	Thorax 2017: 72(1): 57-65.	Randomised, controlled study
Güell M-R, Cejudo P, Ortega F, Puy MC, Rodríguez-Trigo G, Pijoan JI, Martinez-Indart L, Gorostiza A, Bdeir K, Celli B, Galdiz JB.	2017	Benefits of Long-Term Pulmonary Rehabilitation Maintenance Program in Patients with Severe Chronic Obstructive Pulmonary Disease. Three-Year Follow-up.	American Journal of Respiratory and Critical Care Medicine 2017: 195(5): 622-629.	Randomised, controlled study
Do_CBT-based psychological therapies				
Pollok J, van Agteren JEM, Esterman AJ, Carson - Chahhoud KV.	2019	Psychological therapies for the treatment of depression in chronic obstructive pulmonary disease.	Cochrane Database of Systematic Reviews 2019(3)	Cochrane review
Zhang X, Yin C, Tian W, Lu D, Yang X.	2020	Effects of cognitive behavioral therapy on anxiety and depression in patients with chronic obstructive pulmonary disease: A meta-analysis and systematic review.	The Clinical Respiratory Journal 2020: 14(10): 891-900.	Systematic review and meta-analysis
Baraniak A, Sheffield D.	2011	The efficacy of psychologically based interventions to improve anxiety, depression and quality of life in COPD: A systematic review and meta-analysis.	Patient Education and Counseling 2011: 83(1): 29-36	Systematic review and meta-analysis
Coventry PA, Bower P, Keyworth C, Kenning C, Knopp J, Garrett C, Hind D, Malpass A, Dickens C.	2013	The Effect of Complex Interventions on Depression and Anxiety in Chronic Obstructive Pulmonary Disease: Systematic Review and Meta-Analysis.	PLOS ONE 2013: 8(4): e60532.	Systematic review and meta-analysis

Heslop-Marshall K, Baker C, Carrick-Sen D, Newton J, Echevarria C, Stenton C, Jambon M, Gray J, Pearce K, Burns G, De Soyza A.		2018	Randomised controlled trial of cognitive behavioural therapy in COPD	ERJ Open Research 2018: 4(4): 00094-02018.	Randomised, controlled study
Do_supportive care					
Handheld fan	Johnson MJ, Booth S, Currow DC, Lam LT, Phillips JL.	2016	A Mixed-Methods, Randomized, Controlled Feasibility Trial to Inform the Design of a Phase III Trial to Test the Effect of the Handheld Fan on Physical Activity and Carer Anxiety in Patients With Refractory Breathlessness.	J Pain Symptom Manage 2016: 51(5): 807-815.	Feasibility trial
	Barnes-Harris M, Allgar V, Booth S, Currow D, Hart S, Phillips J, Swan F, Johnson MJ.	2019	Battery operated fan and chronic breathlessness: does it help?	BMJ Support Palliat Care 2019: 9(4): 478-481.	A secondary exploratory analysis of 2 randomised controlled trials
	Galbraith S, Fagan P, Perkins P, Lynch A, Booth S.	2010	Does the use of a handheld fan improve chronic dyspnea? A randomized, controlled, crossover trial.	J Pain Symptom Manage 2010: 39(5): 831-838.	Randomised controlled study
	Marchetti N, Lammi MR, Travaline JM, Ciccolella D, Civic B, Criner GJ.	2015	Air Current Applied to the Face Improves Exercise Performance in Patients with COPD.	Lung 2015: 193(5): 725-731.	Crossover study
Nutritional support	Ferreira IM, Brooks D, White J, Goldstein R.	2012	Nutritional supplementation for stable chronic obstructive pulmonary disease.	Cochrane Database of Systematic Reviews 2012(12).	Cochrane review
	Collins PF, Elia M, Stratton RJ.	2013	Nutritional support and functional capacity in chronic obstructive pulmonary disease: A systematic review and meta - analysis.	Respirology 2013: 18(4): 616-629.	Systematic review and meta - analysis
	Jolliffe DA, Greenberg L, Hooper RL, Mathyssen C, Rafiq R, de Jongh RT, Camargo CA, Griffiths CJ, Janssens W, Martineau AR.	2019	Vitamin D to prevent exacerbations of COPD: systematic review and meta-analysis of individual participant data from randomised controlled trials.	Thorax 2019: 74(4): 337-345.	Systematic review and meta-analysis

Do not_ pharmacological management					
SAMA	Cheyne L, Irvin - Sellers MJ, White J.	2015	Tiotropium versus ipratropium bromide for chronic obstructive pulmonary disease.	Cochrane Database of Systematic Reviews 2015(9).	Cochrane review
Routine use of systemic corticosteroids	Walters JAE, Tan DJ, White CJ and Wood - Baker R.	2018	Different durations of corticosteroid therapy for exacerbations of chronic obstructive pulmonary disease.	Cochrane Database of Systematic Reviews. 2018.	Cochrane review
Nebulised opioids	Barnes H, McDonald J, Smallwood N, Manser R.	2016	Opioids for the palliation of refractory breathlessness in adults with advanced disease and terminal illness.	Cochrane Database of Systematic Reviews 2016(3).	Cochrane review
	Ekström M, Nilsson F, Abernethy AA, Currow DC.	2015	Effects of Opioids on Breathlessness and Exercise Capacity in Chronic Obstructive Pulmonary Disease. A Systematic Review.	Annals of the American Thoracic Society 2015: 12(7): 1079-1092.	Systematic review
Theophylline	Wilairat P, Kengkla K, Thayawiwat C, Phlaisaithong P, Somboonmee S, Saokaew S.	2019	Clinical outcomes of theophylline use as add-on therapy in patients with chronic obstructive pulmonary disease: A propensity score matching analysis.	Chronic Respiratory Disease 2019: 16: 1479973118815694.	Observational study
PDE_4	Janjua S, Fortescue R, Poole P.	2020	Phosphodiesterase - 4 inhibitors for chronic obstructive pulmonary disease.	Cochrane Database of Systematic Reviews 2020(5).	Cochrane review
Do not_palliative oxygen					
Abernethy AP, McDonald CF, Frith PA, Clark K, Herndon JE, II, Marcello J, Young IH, Bull J, Wilcock A, Booth S, Wheeler JL, Tulsky JA, Crockett AJ, Currow DC.		2010	Effect of palliative oxygen versus room air in relief of breathlessness in patients with refractory dyspnoea: a double-blind, randomised controlled trial.	The Lancet 2010: 376(9743): 784-793.	Randomised controlled study
Do not_PR during hospitalisation					
Greening NJ, Williams JEA, Hussain SF, Harvey-Dunstan TC, Bankart MJ, Chaplin EJ, Vincent EE, Chimera R, Morgan MD, Singh SJ, Steiner MC.		2014	An early rehabilitation intervention to enhance recovery during hospital admission for an exacerbation of chronic respiratory disease: randomised controlled trial.	BMJ 2014: 349: g4315.	Randomised controlled study

	Tang CY, Blackstock FC, Clarence M, Taylor NF.	2012	Early rehabilitation exercise program for inpatients during an acute exacerbation of chronic obstructive pulmonary disease: a randomized controlled trial.	J Cardiopulm Rehabil Prev 2012: 32(3): 163-169.	Randomised controlled study
Do not_telehealth					
	Gregersen TL, Green A, Frausing E, Ringbæk T, Brøndum E, Suppli Ulrik C.	2016	Do telemedical interventions improve quality of life in patients with COPD? A systematic review.	International journal of chronic obstructive pulmonary disease 2016: 11: 809-822.	Systematic review
	McLean S, Nurmatov U, Liu JLY, Pagliari C, Car J, Sheikh A.	2011	Telehealthcare for chronic obstructive pulmonary disease.	Cochrane Database of Systematic Reviews 2011(7).	Cochrane review
	Cartwright M, Hirani SP, Rixon L, Beynon M, Doll H, Bower P, Bardsley M, Steventon A, Knapp M, Henderson C, Rogers A, Sanders C, Fitzpatrick R, Barlow J, Newman SP.	2013	Effect of telehealth on quality of life and psychological outcomes over 12 months (Whole Systems Demonstrator telehealth questionnaire study): nested study of patient reported outcomes in a pragmatic, cluster randomised controlled trial.	BMJ 2013: 346: f653.	Randomised controlled study
Don't know_ pharmacological management					
SABA	Wilkinson M, Bulloch B, Garcia-Filion P and Keahey L	2011	Efficacy of racemic albuterol versus levalbuterol used as a continuous nebulization for the treatment of acute asthma exacerbations: a randomized, double-blind, clinical trial	Journal of Asthma. 2011; 48: 188-93.	Randomised controlled study
	Jat KR, Khairwa A	2013	Levalbuterol versus albuterol for acute asthma: A systematic review and meta-analysis	Pulmonary Pharmacology & Therapeutics. 2013; 26: 239-48.	Systematic review and meta analysis
	Brunetti L, Poiani G, Dhanaliwala F, Poppiti K, Kang H and Suh D-C	2015	Clinical outcomes and treatment cost comparison of levalbuterol versus albuterol in hospitalized adults with chronic obstructive pulmonary disease or asthma	American Journal of Health-System Pharmacy. 2015; 72: 1026-35.	Randomised controlled study
ICS	Yang IA, Clarke MS, Sim EHA, Fong KM.	2012	Inhaled corticosteroids for stable chronic obstructive pulmonary disease	Cochrane Database of Systematic Reviews 2012(7).	Cochrane review

	Vestbo J, Anderson JA, Brook RD, Calverley PM, Celli BR, Crim C, Martinez F, Yates J, Newby DE, SUMMIT Investigators.	2016	Fluticasone furoate and vilanterol and survival in chronic obstructive pulmonary disease with heightened cardiovascular risk (SUMMIT): a double-blind randomised controlled trial.	The Lancet 2016: 387(10030): 1817-1826.	Randomised controlled study
Antidepressants	Pollok J, van Agteren JEM, Carson‐Chahhoud KV.	2018	Pharmacological interventions for the treatment of depression in chronic obstructive pulmonary disease.	Cochrane Database of Systematic Reviews 2018(12).	Cochrane review
Benzodiazepines	Simon ST, Higginson IJ, Booth S, Harding R, Weingärtner V, Bausewein C.	2016	Benzodiazepines for the relief of breathlessness in advanced malignant and non‐malignant diseases in adults.	Cochrane Database of Systematic Reviews 2016(10).	Cochrane review
	Ekström MP, Bornefalk-Hermansson A, Abernethy AP, Currow DC.	2014	Safety of benzodiazepines and opioids in very severe respiratory disease: national prospective study.	BMJ 2014: 348: g445.	Cohort study
Routine use of mucolytics	Poole P, Sathananthan K, Fortescue R.	2019	Mucolytic agents versus placebo for chronic bronchitis or chronic obstructive pulmonary disease.	Cochrane Database of Systematic Reviews 2019(5).	Cochrane review

Don’t know_relaxation				
Volpato E, Banfi P, Rogers SM, Pagnini F.	2015	Relaxation Techniques for People with Chronic Obstructive Pulmonary Disease: A Systematic Review and a Meta-Analysis.	Evidence-Based Complementary and Alternative Medicine 2015: 2015: 628365.	Systematic review and meta-analysis
Volpato E, Banfi P, Nicolini A, Pagnini F.	2018	A quick relaxation exercise for people with chronic obstructive pulmonary disease: explorative randomized controlled trial.	Multidisciplinary Respiratory Medicine 2018: 13(1): 13.	Randomised controlled study
Don’t know_breathing exercises				
Holland AE, Hill CJ, Jones AY, McDonald CF.	2012	Breathing exercises for chronic obstructive pulmonary disease	Cochrane Database of Systematic Reviews 2012(10).	Cochrane review

Ubolnuar N, Tantisuwat A, Thaveeratitham P, Lertmaharit S, Kruapanich C, Mathiyakom W.	2019	Effects of Breathing Exercises in Patients With Chronic Obstructive Pulmonary Disease: Systematic Review and Meta-Analysis.	Ann Rehabil Med 2019: 43(4): 509-523.	Systematic review and meta-analysis
Borge CR, Hagen KB, Mengshoel AM, Omenaas E, Moum T, Wahl AK.	2014	Effects of controlled breathing exercises and respiratory muscle training in people with chronic obstructive pulmonary disease: results from evaluating the quality of evidence in systematic reviews.	BMC Pulmonary Medicine 2014: 14(1): 184.	Systematic review
